# A performing arts intervention to decrease suicide stigma on campus: A three time point assessment of “Every Brilliant Thing”

**DOI:** 10.1371/journal.pmen.0000404

**Published:** 2025-08-14

**Authors:** Orrin D. Ware, Mimi V. Chapman, Denise Yookong Williams, Vivienne Benesch, Jeff Aguiar

**Affiliations:** 1 School of Social Work, University of North Carolina at Chapel Hill, Chapel Hill, North Carolina, United States of America; 2 PlayMakers Repertory Company, University of North Carolina at Chapel Hill, Chapel Hill, North Carolina, United States of America; PLOS: Public Library of Science, UNITED KINGDOM OF GREAT BRITAIN AND NORTHERN IRELAND

## Abstract

Suicidality is stigmatized, with discussions on the topic considered taboo by some. Fine arts may tackle subjects that people find hard to address. In 2024, a tour of “Every Brilliant Thing,” an interactive performance that introduces the topics of suicidality and suicide loss, was held on a university campus. During campuswide performances, attendees were recruited to complete anonymous self-report surveys that captured the Stigma of Suicide Scale Short Form at: Pre-Performance, Post-Performance, and 30-Day Follow-Up. Analysis of Variance with Bonferroni test for post hoc analysis examined differences in the stigma of suicide between the three timepoints. There were 196 responses at Pre-Performance, 151 responses at Post-Performance, and 104 responses at 30-Day Follow-Up. Most of the sample self-identified as female/woman (77.6%) and White (82.7%). A slight mean decrease was identified in scores for the Stigma of Suicide Scale Short Form: Overall and the Stigma of Suicide Scale Short Form: Stigma Subscale from pre-performance to post-performance. A slight decrease in the Stigma of Suicide Scale Short Form: Overall was also identified from the pre-performance timepoint to the 30-day follow-up timepoint. Considering the lifesaving importance of addressing the stigma of suicide and the innumerable persons in need of services, the fine arts can be an essential tool to reduce stigma.

## Introduction

Suicide is a prominent public health concern. In 2022, in the U.S., 13 million adults experienced suicidal ideations, and 3.8 million reported making a suicide plan [1]. Approximately 1.6 million people attempted suicide, and approximately 50,000 people died by suicide during that timeframe [1,2]. When death by suicide happens on college campuses the impacts are substantial. Suicide diffusion, in which suicide attempts may cluster and occur within a distinct location or time period, pose an on-going risk after a death of this nature [3–6]. Students, faculty, and staff may experience difficulties in continuing with learning and other campus functioning. In the event of a suicide, administrators are often tasked with the difficult role of navigating nuanced suicide postvention issues related to privacy and safe messaging within the constraints of distinct and, at times, conflicting individual and community needs. In some cases, a family’s desire for privacy may be prioritized over a community’s need for conversation. These realities create potential situations in which talking about suicide becomes further stigmatized during a time when a campus community arguably most needs support and opportunities for open conversations related to mental health and suicide.

Treatments such as counseling and pharmacotherapy are options that exist for individuals experiencing severe suicidal ideations or who may be at risk of suicide [7–12]. Yet, suicide prevention efforts are harder to infuse into the public sphere. Despite the availability of treatments and interventions, discussion of suicide is often considered taboo. Long-held beliefs that talking about suicide is tacit encouragement of such behavior contribute to stigmatization around suicidality conversations, even as clinicians recognize that such conversations may actually save lives [13,14]. Consequently, campuses have struggled with how to incorporate suicide prevention and suicide stigma reduction into wider mental health efforts [15–17]. Innumerable persons in need of suicide prevention services may not access them due to the stigma of suicide, ranging from multifaceted layers of public stigma, personal suicide stigma, and self-stigma [18]. Suicide stigma may leave a void in which many persons are reluctant to discuss mental health challenges or suicidal thoughts and behaviors, further limiting their potential access to interventions, treatments, or other services. In fact, studies have shown that both public stigma and self-stigma are associated with lower willingness to seek help for mental health issues, especially related to suicide [19,20]. Considering the prevalence of suicide and the barrier that stigma places on individuals needing suicide prevention services, it is imperative to implement strategies that reduce the stigma of suicide.

Since prehistoric times, humans have continued engaging with the arts to foster development, culture, and consciousness, and art has played an imperative role in rituals across cultures worldwide for ages [21]. The field of expressive art therapy itself has grown in popularity as it draws from various theoretical perspectives including foundational psychodynamic processes and symbolism, in conjunction with more nascent theoretical underpinnings such as adaptive response theory, to promote meaning-making, communication, self-awareness, healing, and growth [22,23]. The arts, including visual, literary, and performing arts, have long tackled subjects that people find hard to address in daily life. Examples include child maltreatment, domestic violence, discrimination, disability, and mental health. Literature, both fiction and non-fiction, has served to raise public consciousness about difficult sociological issues as well as interpersonal and psychological difficulties. Visual and performing arts create the same opportunity for increased conversation and decreased stigma for topics that are polarizing or sometimes deeply shameful. A growing body of research suggests that artistic self-expression and interventions can yield promising results, especially for stigmatized subjects and among harder-to-reach populations [24–26]. Chapman and colleagues found changes in levels of explicit and implicit bias toward new immigrant populations and decreases in anti-Muslim bias using arts-based interventions [27]. Recently, Pinto and colleagues used illustrative images to facilitate critical dialogues to increase understanding of COVID-19 prevention practices [28]. In thinking about suicide specifically, the Netflix show ‘Thirteen Reasons Why’ was associated with reduced stigma around suicidal ideations and higher odds of discussing self-harm [29]. Another example includes researchers using theater to address the stigma associated with mental health disorders [30].

Multiple systematic and scoping reviews of arts-based interventions document utility and evidence supporting the use of the arts in promoting mental health initiatives, facilitating dialogue, increasing empathy, decreasing stigma, and enhancing protective measures against suicidality [26,31,32]. More specifically, growing evidence shows positive impacts of community-based theater suicide prevention interventions in addressing suicidality by decreasing known risk factors (e.g., suicide-related stigma) and increasing life promotive factors (e.g., mental health awareness, knowledge of resources, individual empowerment, and self-efficacy in help-seeking behaviors in the event of suicidal ideation), especially across cultures [19,33–36]. Because college campuses often have a wealth of artistic resources on campus, making use of those resources to address campus well-being and suicidality, in particular, is worth considering [37,38].

In 2024, PlayMakers Repertory Company [39], a professional theater in residence at the University of North Carolina at Chapel Hill; implemented a campuswide tour of “Every Brilliant Thing,” an interactive performance by Duncan Macmillan with Johnny Donahoe that introduces the topics of mental health disorders, suicidal ideations, and suicide loss from the perspective of the child of someone who had these experiences [40,41]. These performances ran from January 10^th^ 2024, to February 21^st^ 2024 [42]. Performances were held across the campus so that students in all campus units had a chance to participate. All campus affiliates attended free of charge. After the performance concluded, facilitated post-show discussions were held as an open space for attendees to discuss and process their thoughts. Post-show discussions were held immediately after the performance and were optional. A semi-structured discussion guide was created by the performance implementation team. In addition, mental health first aid personnel were available to anyone wanting immediate assistance. Likewise, information about mental health resources on campus was provided. The current study examined the stigma of suicide among audience members at three time points: before, after, and at 30-day follow-up after participating in a performance of “Every Brilliant Thing” [40,41]. Since the performance offers an interactive experience that allows audience members to experience empathy, this study focused on examining the potential impact on stigma. Empathy is often regarded as a critical component in reducing stigma towards mental health [43,44]. Other secondary outcomes were also examined in this study, such as suicide Isolation/Depression and suicide Glorification/Normalisation. However, the primary research aim proposed in this study was to identify whether reductions in suicide stigma would be observed after participants watched “Every Brilliant Thing” during a campus-wide tour of the performance. There were no a priori hypotheses for this study.

## Materials and methods

### Ethics statement

All study procedures were approved by the University of North Carolina at Chapel Hill Institutional Review Board (IRB; IRB Number: 23–2496; Principal Investigator (PI): Ware). In accordance with the University of North Carolina at Chapel Hill IRB determination, data collected from this study cannot be shared with anyone outside of the IRB-approved research team. A waiver of written (signed) informed consent was approved by the University of North Carolina at Chapel Hill Institutional Review Board in that the research presented no more than minimal risk to potential participants. Instead, potential eligible participants could access a study information page and provide a binary “Yes” or “No” response option for their interest in study participation. More details are provided below in the Recruitment section.

### Recruitment

Blending inquiry and research design into the production process, including outreach efforts, focused considerations around invitation to performances as well as the subject matter. Parallel processes shaped different dimensions of the ask to participate in the study, one administratively and the other creatively in the rehearsal room, reinforcing Block’s assertion that “invitation is more than just a request to attend; it is a call to create an alternative future, to join the possibility we have declared” (p. 112) [45].

Recent trends in arts marketing provide an opportunity for transparency and informed decision-making. Both messaging and community buy-in were critical to framing the moment of invitation offered to audiences before each performance. Accomplishing this required campus-wide collaboration which started with Student Affairs across professional and academic units, growing to include unit-specific collaborators supporting the implementation of performances at their various sites. This collaborative core evaluated general needs for outreach and communication as well as logistical needs to ensure a smooth and cohesive audience experience. This planning began around six months prior to public performances, offering rich opportunities to explore various nuances across prospective participant communities within the university context.

In addition to considerations around getting prospective participants to each performance, Block’s concept of invitation required further examination and extension into both rehearsal and performance processes. Rehearsals became sites for public exploration and consideration as a function of inviting key stakeholders—administrators, faculty, and staff—into the implementation process, creating a foundation for the upcoming collaboration through first-hand experience with the process and the product. In many ways, demonstrating the ways that we would tackle the stigma through performance was an essential component of expanding the reach of word of mouth; that people felt the show could make a difference was an important framing for the beneficence of the study. These points of connection extended the capacity of working teams to explore the intersections of their professional work with their personal care for affected populations.

Opening the rehearsal process to visitors reinforced additional needs in crafting a comprehensive and caring invitation to the research study. Theorizing the experience of a prospective audience participant was crucial to developing strategies that would support and illustrate dialogue as an active social dynamic. In addition to printed and digital resources made available at each performance through the university’s Mental Health First Aid (MHFA), Counseling and Psychological Services, and a peer network collaborative focused on mental health, as well as a local chapter of the National Alliance on Mental Illness, structural and experiential choices were made to support participant experiences and decision-making.

Operationalizing the invitation to participate included the addition of a volunteer team of rapid response agents, comprised of MHFA-certified volunteers and clinicians, who could provide support to individuals overwhelmed by the performance experience in real-time. Each performance also included a post-show discussion often facilitated by a team comprised of a member of the artistic team and a mental health clinician, utilizing a facilitation guide developed in collaboration with MHFA. Audiences were also offered an anonymous opportunity to participate in a community-building activity whenever they exited the space. These considerations created multiple points-in-time where prospective participants might see themselves in some type of social dialogue with varying degrees of risk and vulnerability.

Specific to the survey, individuals attending the performance were provided a playbill. Inside the playbill was a study flyer that contained [a] a brief description, [b] a QR code, [c] a link, [d] University of North Carolina at Chapel Hill IRB contact information, and [e] contact information for the study’s PI. Before the performance, a representative of PlayMakers Repertory Company provided attendees with a brief description of the study and described how they could use the flyer to access the study.

Using the link or QR code found on the flyer would take participants to a study information page for the “Every Brilliant Thing” Evaluation Study in Qualtrics, a platform that allows users to create surveys and distribute, collect, and analyze response data [46]. The study information page described the study’s purpose as learning more about people’s perspectives on mental health. The incentive was described as a drawing that was based on chance, where five randomly selected individuals who completed any of the three surveys would receive a $50 virtual gift card. Study procedures were described as voluntary 5–10-minute surveys at three different time points. At the end of the study information page was a binary “Yes” or “No” response option for their interest in study participation. A “No” response exited the survey, and a “Yes” response continued the survey. A question, “What is your age in years?” had one category, “Younger than 18 years old,” that, if selected, would identify an individual as being ineligible for the study.

The study included three time points, pre-performance, post-performance, and thirty-day follow-up. However, individuals were not tracked across time points. Instead, responses were aggregated across the three groups (pre-performance, post-performance, and thirty-day follow-up), which allowed for increased confidentiality for our respondents. Pre-performance and post-performance surveys were differentiated by the following question: “Did you watch the performance of “Every Brilliant Thing” at the University of North Carolina at Chapel Hill? Please select “No” if you are a current attendee, and the performance has not started or concluded.” “No” responses to this question were included in the pre-performance group, and “Yes” responses were included in the post-performance group.

After completing either the pre- or post-performance survey, participants could opt in to provide their e-mail address to “Complete a follow-up survey approximately thirty days after watching the performance.” These thirty-day follow-up surveys were sent to respondents 30 days (+/- 4 days) after they completed the survey. Via Qualtrics [46] e-mails were sent with a description of the follow-up survey including [a] a brief description, [b] a QR code, [c] a link for the survey, [d] University of North Carolina at Chapel Hill Institutional Review Board contact information, and [e] contact information for the study’s PI. Individuals who did not complete the follow-up survey in five days of the e-mail were sent a reminder e-mail. Surveys that were completed via the follow-up e-mails were categorized as “30-Day Follow-Up.” Incentives were dispensed after collecting the thirty-day follow-up data. Study participants could opt in to receive an e-mail containing mental health resources on campus; this information was also dispensed after collecting the thirty-day follow-up data. Initial IRB approval for this study was received on October 20, 2023. Data were captured from the first day of the performance on January 10, 2024, with follow-up data being captured until April 13, 2024.

### Measures

#### Timepoint.

As described in the Recruitment section above, surveys were categorized into a trinary variable with the following values: [a] Pre-Performance, [b] Post-Performance, and [c] 30-Day Follow-Up. Surveys with a “No” to watching the performance were categorized as “Pre-Performance” while surveys with a “Yes” response were categorized as “Post-Performance”. Surveys that were completed via e-mails sent at the 30-Day Follow-Up period after the performance were categorized as “30-Day Follow-Up”.

#### Age.

Age in years was captured by the following question, “What is your age in years?” Respondents would select from a dropdown that ranged from 18 to 75. Two categorical values were also present, “younger than 18 years old” and “older than 75 years old”. Individuals who selected “younger than 18 years old” were ineligible to participate in the study and were unable to complete the survey. Any responses that selected “older than 75 years old” were recoded to 76 to use age as a continuous variable while including outliers.

#### Gender.

Gender is a categorical variable captured with the following question, “How do you describe yourself? (select one).” Response options include [a] Female/woman, [b] Male/man, [c] Genderqueer, gender non-conforming, or non-binary, [d] Transgender male/man, [e] Transgender female/woman, and [f] Different identity. Because of small cell sizes, some responses were grouped into the category “Another Gender Identity” when reporting study results.

#### Ethnicity.

Ethnicity is a binary variable with [a] Hispanic or Latino and [b] Non-Hispanic or Latino as response options to the following question, “What best describes your ethnicity?”

#### Race.

Race included six binary variables as a response to the following question, “What best describes your race (please select all that apply)?” Selecting any of the following indicated that the individual identified as that race, [a] American Indian or Alaska Native, [b] Asian, [c] Black or African American, [d] Native Hawaiian or Other Pacific Islander, [e] White, and [f] Other (please describe). These responses were not mutually exclusive, and an individual could select any or all of these categories. Because of small cell sizes, some responses were grouped into the category “Another Race” when reporting study results.

#### Affiliation.

Affiliation included ten binary variables as a response to the following question, “What is your affiliation with the University of North Carolina at Chapel Hill (please select all that apply)?” Selecting any of the following indicated that the individual identified with that affiliation, [a] Alumni, [b] Current undergraduate student, [c] Current graduate or professional student (including JD, Maryland, PharmD, and PhD student), [d] Current fellow, postdoctoral fellow, or resident, [e] Donor, [f] Employee: Administrator, [g] Employee: Faculty, [h] Employee: Staff, [i] Other (please describe), and [j] None of the above. Similar to the race variable, these responses were not mutually exclusive. Due to small cell sizes, some responses were grouped into the category “Another Affiliation” when reporting study results.

#### Stigma of suicide scale short form.

The Stigma of Suicide Scale Short Form (SOSS-SF) is a sixteen-item scale with three subscales: [a] Stigma, [b] Isolation/Depression, and [c] Glorification/Normalisation [47,48]. Higher scores on the SOSS-SF indicate greater stigmatization towards individuals who die by suicide [47,48]. Using a Likert scale ranging from strongly disagree (1) to strongly agree (5), respondents identified how much they agreed with descriptions of individuals who die by suicide. Examples of these descriptions include “pathetic” and “shallow” [47,48]. Scores were averaged for the overall measure and for each of the three subscales. This measure was selected as it is a relatively brief way to capture the stigma of suicide, especially considering that respondents are being asked to complete this measure pre-performance. Although there was no a priori hypothesis regarding potential changes in each subscale, by using the SOSS-SF, we were able to examine the potential for change scores with isolation/depression and glorification/normalisation alongside the stigma subscale.

### Analyses

IBM SPSS [49] was used to analyze the study data that were exported from Qualtrics [46]. The initial data export included 487 responses, of which 36 were missing data from the study’s dependent variable measure SOSS-SF. Little’s test of missing completely at random (MCAR) was conducted to determine whether listwise deletion or multiple imputation should used to address the missing data [50,51]. Since Little’s MCAR was non-significant (*p* > .05), we performed listwise deletion and analyzed a final sample of **N* *= 451. Univariable statistics such as means, percentages, and standard deviations were used to examine the study sample. To ensure further protection for study participants, IRB-approved study procedures require aggregating any group with less than 20 respondents. Analysis of Variance (ANOVA) was used to examine differences in SOSS-SF between the three timepoints, [a] Pre-Performance, [b] Post-Performance, and [c] 30-Day Follow-Up. Bonferroni test was used as a post hoc analysis of the mean differences, and Eta-squared was examined for effect sizes. Results were considered significant if *p* < .05.

## Results

A description of the study sample may be seen in Table 1. The majority of the sample self-identified as female/woman (77.6%), non-Hispanic or Latino (94.9%), and White (82.7%). There were 196 responses for the Pre-Performance timepoint, 151 responses for the Post-Performance timepoint, and 104 responses for the 30-Day Follow-Up timepoint. The Cronbach alpha for the SOSS-SF for the total sample and three different timepoints may be found in Table 2. Further, means and standard deviations for the SOSS-SF may be found in Table 2.

**Table 1:** Descriptives of the study sample, N = 451.

	n	%
**Sample Size**	451	100.0
**Timepoint**		
Pre-Performance	196	43.5
Post-Performance	151	33.5
30-Day Follow-Up	104	23.1
**Age in years, Mean (SD)** ^**1**^	41.9 (18.4)	
**Gender**		
Female/woman	350	77.6
Male/Man	78	17.3
Another Gender Identity	23	5.1
**Ethnicity**		
Hispanic or Latino	23	5.1
Non-Hispanic or Latino	428	94.9
**Race**		
Asian	45	10.0
Black or African American	29	6.4
White	373	82.7
Another Race	20	4.8
**University Affiliation**		
Alumni	78	17.3
Current Graduate or Professional Student	50	11.1
Current Undergraduate Student	89	19.7
Employee: Staff	60	13.3
Another Affiliation	128	28.4

Note: To ensure further protection for study participants,

IRB-approved study procedures require aggregating any group with less than 20 respondents.

^1^“older than 75 years old” were recoded to 76 to use age as a continuous variable while including outliers

**Table 2:** Cronbach’s alpha and means the stigma of Suicide Scale Short Form (SOSS-SF).

	Total Sample^1^		Pre-Performance^2^		Post-Performance^3^		30-Day Follow-Up^4^	
	Cronbach alpha	Mean (SD)	Cronbach alpha	Mean (SD)	Cronbach alpha	Mean (SD)	Cronbach alpha	Mean (SD)
SOSS-SF Overall	.770	2.3 (0.4)	.772	2.4 (0.4)	.755	2.3 (0.4)	.772	2.3 (0.4)
SOSS-SF: Stigma Subscale	.916	1.4 (0.6)	.921	1.5 (0.6)	.913	1.3 (0.5)	.899	1.3 (0.5)
SOSS-SF: Isolation/Depression Subscale	.879	3.9 (0.8)	.880	3.9 (0.8)	.870	3.8 (0.8)	.896	3.9 (0.8)
SOSS-SF: Glorification/Normalization Subscale	.857	2.7 (0.7)	.814	2.7 (0.6)	.890	2.7 (0.8)	.863	2.6 (0.8)

^1^N = 451

^2^n = 196

^3^n = 151

^4^n = 104

One-way ANOVA identified significant differences in mean scores for the SOSS-SF Overall (*F*(2, 448)=[4.575], *p* = .011) and the SOSS-SF: Stigma Subscale (*F*(2, 448)=[3.724], *p* = .025). The ANOVA with the SOSS-SF: Isolation/Depression and SOSS-SF: Glorification/Normalisation subscales were non-significant (**p* *> .05). Bonferroni test found mean differences in the SOSS-SF Overall between the pre-performance group and the post-performance group (*p* = .029, 95% Confidence Interval [CI]= 0.008, 0.208). Bonferroni test also identified mean differences in the SOSS-SF Overall between the pre-performance group and the 30-day follow-up group (*p* = .0046, 95% CI = 0.001, 0.225). Regarding the SOSS-SF: Stigma Subscale differences were identified between the pre-performance group and the post-performance group (*p* = .047, 95% CI = .002, 0.300). A power analysis of the ANOVA was completed using data from the Stigma of Suicide Scale and the number of responses for the three timepoints. The analysis indicated a power value of 0.755 and an effect size of 0.167. Examining Eta-squared for each ANOVA, these were 0.020 (95% CI = 0.001, 0.050) for the SOSS-SF Overall, 0.016 (95% CI = 0.000, 0.044) for the SOSS-SF: Stigma subscale, 0.001 (95% CI = 0.000, 0.010) for the SOSS-SF: Isolation/Depression subscale, and 0.005 (95% CI = 0.000, 0.022) for the Glorification/Normalisation subscale. Essentially, the Eta-squared for each ANOVA was small [52], meaning the differences in scores by group were small.

## Discussion

This study adds to the growing evidence demonstrating how arts-based methods can be used to prevent mental health challenges and promote help-seeking behavior. In addition, the paper demonstrates the utility of cross-disciplinary collaboration and university leadership in doing this work. In the case of Every Brilliant Thing, the campus repertory company and department of dramatic art garnered support from the Office of the Dean of Students to make this a campus-wide effort. The School of Social Work was instrumental in both providing the evaluation component and also in providing mental health support during and after the performances.

Findings from this study identified a decrease in suicide stigma from pre-performance to both post-performance and 30-day follow-up. Although this decrease was significant, it was small which points to two essential considerations. First, any reduction in the stigma of suicide, even slight, is a favorable outcome. Second, it shows the importance of using a spectrum of interventions to modify stigma and suicidal behavior. Other findings from this study for the secondary outcomes include no identified effect of the performance on the Isolation/Depression and Glorification/Normalisation subscales. Future studies that incorporate open-ended responses are needed to truly capture how attendees viewed suicide Isolation/Depression and Glorification/Normalization at the three different timepoints.

Often, arts-based approaches focus solely on treatment once a mental health difficulty or crisis has occurred. This study demonstrates the power of the arts in prevention efforts, something less well-studied. Considering the multifaceted nature of stigma related to suicide, efforts across multiple fronts are needed to reduce the multiple layers of suicide stigma, including public stigma, personal suicide stigma, and self-stigma [18]. Stigma, both internal and external, reduces the likelihood of individuals seeking needed services for mental health disorders or self-harm [19,20]. Further, stigma is a potential barrier to having discussions about suicide. Considering there were approximately 50,000 people who died by suicide in 2022 [2], any reduction in suicide stigma, no matter how slight, should be regarded as a public health benefit. Therefore, large-scale arts-based suicide prevention and stigma-reduction efforts are needed, which may address this prominent public health concern.

Largescale arts-based interventions can be driven by concepts such as narrative transportation to capture the audience while addressing topics such as suicide stigma. Narrative transportation describes a psychological state of individuals being steeped into a story, essentially being so immersed that they are fundamentally changed [53–55]. In this study, narrative transportation was likely instrumental in the reductions in the observed suicide stigma since “Every Brilliant Thing,” introduces mental health disorders, suicidal ideations, and suicide loss from the perspective of the child of someone who had these experiences [40,41]. The ability to empathize with someone who had a parent with these experiences may remain with a performance attendee for quite some time.

Along with narrative transportation, which describes audience members being steeped in a story, the collective nature of the theater can cause individuals to experience emotions that extend beyond the self. Collective effervescence, a concept introduced by Emile Durkheim [56], describes a group experiencing common emotions through participation in a shared activity, event, or ritual [57,58]. Since “Every Brilliant Thing,” is interactive in that audience members are not mere observers but also participate in the performance [40,41], the commonality of these experiences can foster collective effervescence [57,58]. By experiencing the collective nature of self-transcendent emotions during the performance, such as care, empathy, and sympathy, as the performer engages with the audience and introduces mental health disorders, suicidal ideations, and suicide loss from the perspective of the child of someone who had these experiences, stigma of suicide may reduce for audience members.

The reduction in the stigma of suicide being maintained not only during the immediate post-performance period but also during the 30-day follow-up period is also a positive outcome. With pre and immediate post-surveys, there is always the risk that the effects of an intervention may not remain beyond the post-surveys. However, our findings identified a similar mean and standard deviation in the stigma of suicide at the post-performance and 30-day follow-up. Overall, findings from this study suggest that among our sample of adults who watched a live performance of “Every Brilliant Thing” [40,41], on a university campus, reductions in the stigma of suicide may be identified. Considering the lifesaving importance of addressing the stigma of suicide and the innumerable persons in need of services, using the arts as a gateway to increase dialogue, increase awareness, and reduce stigma is imperative.

## Conclusion

This study is the first to report findings related to the stigma of suicide at three different timepoints among attendees watching a live performance of “Every Brilliant Thing” on a university campus. Although decreases in suicide stigma were slight, this initial study set the stage for future studies examining qualitative data captured from participants to yield more robust perspectives of the potential impact of attending these performances. Overall, although the decreases in suicide stigma were slight as identified in this study, any reduction may be viewed as beneficial to address the pressing public health concern of suicidality.

## Limitations

Findings from this study, which examined the stigma of suicide before and after attending a performance of “Every Brilliant Thing” [40,41] should be considered alongside limitations. Individual respondents were not tracked across each separate timepoint as the respondents were aggregated. This is consistent with study procedures approved by the University of North Carolina at Chapel Hill Institutional Review Board. The authors acknowledge that this is a methodological limitation in that we cannot assess the changes in individual scores across time, which may impact the study’s internal validity. Instead, this study can only report aggregated scores across the three timepoints, which is not the same as examining the scores of individuals. Also consistent with the University of North Carolina at Chapel Hill Institutional Review Board approved study procedures, to increase the anonymity of the data further, we were unable to present descriptive characteristics of the three separate timepoints. Future studies with more robust samples across the different timepoints may analyze individual and group changes across different data collection timepoints. Although the survey was brief, approximately 5–10 minutes, individuals responding to the pre-performance survey may have completed the survey quickly to watch the performance which may have biased their responses. Reflecting on reporting aggregate scores even among subgroups may bias study findings. Self-selection bias, where individuals who choose to participate in a study may have different characteristics compared to those who do not, may have a direct impact on this study’s results [59,60]. As we are unable to describe the respondent characteristics such as age, gender, ethnicity, race, and university affiliation across the three different timepoints, we are unable to assess how subgroup participation varied. Another limitation is individuals who may opt to watch the performance may already have lower stigma towards suicide.
